# Responding to extreme stress: Resilience and psychological distress in undergraduate nursing students in the Western Cape, South Africa

**DOI:** 10.4102/curationis.v49i1.2845

**Published:** 2026-06-20

**Authors:** Jennifer Chipps, Regis R. Marie Modeste, Amanda Cromhout, Ilze Steenkamp

**Affiliations:** 1 School of Nursing, Faculty of Community and Health Sciences, University of the Western Cape, Cape Town, South Africa; 2 Department of Nursing and Midwifery, Faculty of Health and Wellness Sciences, Stellenbosch University, Cape Town, South Africa

**Keywords:** COVID-19, psychological distress, resilience, nursing students, mental health, South Africa

## Abstract

**Background:**

Nursing students face significant academic and clinical stressors that can affect their psychological well-being. These challenges are intensified during extraordinary events, such as the coronavirus disease 2019 (COVID-19) pandemic.

**Objectives:**

This study investigated psychological distress and resilience among junior (1st year – 2nd year) and senior (3rd year – 4th year) undergraduate nursing students exposed to a major stressor at two universities in the Western Cape, South Africa.

**Method:**

A survey was conducted among 589 nursing students during and after COVID-19. Most respondents were female (77.4%) and junior students (75.2%), with a mean age of 22.5 years (± 4.6). Data collection took place at University A (February 2021–March 2021) and University B (November 2021–February 2022). Measures include psychological distress (Kessler-10), resilience (Response to Stressful Experiences Scale) and fear of COVID-19 (Fear of COVID-19 Scale).

**Results:**

Overall, students reported mild psychological distress and fear of COVID-19, and high levels of resilience. Senior students experienced significantly higher psychological distress than juniors (*p* < 0.001), but lower fear of COVID-19 (*p* = 0.028). Resilience did not differ significantly between groups, with most students demonstrating high resilience.

**Conclusion:**

Nursing students showed strong resilience despite the added pandemic-related stressors. Elevated distress among seniors, however, highlights the need for targeted psychological support. Strengthening resilience within nursing education may prepare students to manage future crises and contribute to a sustainable, adaptable nursing workforce.

**Contribution:**

This study provides evidence on the psychological well-being of nursing students in South Africa during the COVID-19 pandemic. It emphasises the importance of resilience-building interventions within nursing curricula and institutional support systems to safeguard student health and professional readiness.

## Introduction

Nursing students face a variety of stressors, both academically and clinically. Academically, students must cope with heavy workloads, academic expectations, pressure to succeed, and decisions about possible career trajectories (Beiter et al. 2015). Clinically, students may be overwhelmed by the number of weekly clinical hours. They may also fear making mistakes and harming patients (Mohamed et al. 2024), feelings of incompetence as a result of lack of experience, and having to manage the expectations of staff, patients and families of patients (Dias et al. 2024).

Furthermore, these stressors may manifest differently in junior and senior nursing students because of several factors. Senior students have higher workloads than junior students and more responsibility in clinical placement. Clinical exposure for nursing students becomes increasingly complex as they advance through their years of study. In their first and second years, students mainly engage in foundational, non-clinical activities such as community-based nursing, health promotion and preventative care. In contrast, 3rd year and 4th year students gain experience in various healthcare environments, including primary, secondary and tertiary care settings. During this time, as documented by Mafumo and Netshikweta (2022), they take on more advanced clinical responsibilities (e.g. suturing, monitoring labour and conducting deliveries, catheterisation, inserting a nasogastric tube; writing off duties and ordering supplies) and technical procedures that match their level of training and competence. Lavoie-Tremblay et al. (2022) found that stressors among undergraduate nursing students from Canada mainly related to the specific novelty of the academic year: first year nursing students mainly experienced academic-related stress (e.g. course workloads, overlapping assignment deadlines and exam preparation), while second year students’ stress mainly stemmed from academic (e.g. high academic workloads), clinical (e.g. having to demonstrate skills) and personal (e.g. not having enough time for personal life) sources. Third year students were mainly concerned about graduating and transitioning to the work environment, and final year students about writing their licencing exam. In South Africa, final-year nursing students often express concern about their placement for mandatory community service, a government-regulated programme that requires newly qualified nurses to complete a year of supervised work in public healthcare facilities. This initiative, formalised in Government Gazette Notice No. 765 of 24 August 2007, is designed to ensure equitable healthcare delivery across the country while providing graduates with practical experience before full professional registration.

In addition, stress levels among nursing students often increase during periods of heightened or prolonged pressure. For example, while their training includes preparation for managing infectious diseases, the emergence of an unfamiliar outbreak, such as the coronavirus disease 2019 (COVID-19) pandemic, introduced additional psychological and academic stressors (Feyzbabaie et al. 2025). These challenges included the sudden transition to online learning, limited internet connectivity, unfamiliarity with digital platforms and tools (Ashipala, Mathias & Shikukumwa 2023), the lack of standardised distance education frameworks, and difficulties studying in distracting home environments (Masha’al, Rababa & Shahrour 2020). Additionally, nursing students faced significant challenges during clinical placements, which included fears of contracting the virus (Ulenaers et al. 2021), and widespread shortages of essential resources (Mbunge 2020; Nyasulu & Pandya 2020).

These challenges can lead to psychological problems, such as fear, anxiety and depression (Mbunge 2020); poor academic performance (Gallego-Gómez et al. 2020); and maladaptive coping strategies, such as substance abuse and binge eating (Savitsky et al. 2020; Subba et al. 2020). However, studies have shown that although nursing students reported higher levels of stress and anxiety during the pandemic than pre-pandemic, they still only reported moderate to high levels of stress and anxiety, and moderate to high levels of resilience (Labrague 2021; Savitsky et al. 2020). While higher levels of resilience are associated with lower levels of stress and anxiety (Labrague 2021; Savitsky et al. 2020); not having sufficient resources, and fear of infection were associated with higher levels of anxiety (Savitsky et al. 2020).

### Aim of the study

The aim of this study was to investigate psychological distress and resilience in undergraduate nursing students from two universities in the Western Cape, South Africa.

## Research methods and design

### Research design and study population

A survey design was used to investigate psychological distress and resilience among undergraduate nursing students at two adjacent universities in the Western Cape, South Africa. The study focused on junior (1st year and 2nd year) and senior (3rd year and 4th year) students, while postgraduate students, already qualified and practising nurses, were excluded. With an approximately 1400 population size, using sample size calculator with consideration of 99% confidence level and 4% margin of error and 50% population proportion, the sample size was planned at 564. Through convenience sampling, a total of 589 participants were included in the final sample, comprising 380 respondents from University A and 209 respondents from University B. Both institutions are situated in the same geographic area, with a student population of approximately 1000 and 400, respectively. They offer the Bachelor of Nursing programme and share access to the same clinical training facilities.

### Instruments

A self-administered questionnaire was used to collect demographic information (age, gender and year of study) and three validated scales to measure stress and resilience.

Psychological distress was assessed using the Kessler Psychological Distress Scale (Kessler-10) (Kessler et al. 2002), which measures distress experienced during the past 30 days. The scale consists of 10 items rated on a 5-point Likert scale (1 = none of the time to 5 = all of the time). Total scores range from 10 to 50, with higher scores indicating greater distress. Based on cut-offs, respondents were classified as ‘likely to be well’ (< 20), mild distress (20–24), moderate distress (25–29) or severe distress (≥ 30). Kessler et al. (2002) reported a Cronbach’s alpha of 0.93; in this study reliability was 0.90 (total sample), 0.88 (junior students) and 0.91 (senior students).

Resilience was measured using the Response to Stressful Experiences Scale (Johnson et al. 2011). This 22-item instrument assesses five domains of coping: (1) active coping, (2) cognitive flexibility, (3) meaning-making, (4) self-efficacy and (5) spirituality. Items are rated on a 5-point Likert scale (0 = not at all like me to 4 = exactly like me). Total scores range from 0 to 88, with higher scores reflecting greater resilience. Respondents were classified as having low (< 49), moderate (50–70) or high (71–88) resilience. Johnson et al. (2011) reported Cronbach’s alpha values between 0.91 and 0.93; in this study, internal consistency was 0.91 (total sample), 0.90 (junior students) and 0.93 (senior students).

The major stressor was the impact of COVID-19, and the fear of COVID-19 was measured using the Fear of COVID-19 Scale (FCV-19S) (Ahorsu et al. 2022). This 7-item scale uses a 5-point Likert response format (1 = strongly disagree to 5 = strongly agree). Scores range from 7 to 35, with higher values indicating greater fear. Respondents were categorised as having mild (7–19), moderate (20–26) or severe (≥ 27) levels of fear (Faro et al. 2022). Ahorsu et al. (2022) reported a Cronbach’s alpha of 0.92; in this study, reliability was 0.87 (total sample), 0.85 (junior students) and 0.90 (senior students).

### Data collection

At University A, data were collected using a self-administered questionnaire conducted in February 2021 and March 2021, prior to the return of first-year and second-year nursing students to clinical practice to complete the practical component of their training. This period coincided with the peak of the COVID-19 pandemic in South Africa, during which adjusted alert level 3 and level 1 were in effect. At University B, questionnaires were administered between November 2021 and February 2022, during adjusted alert level 1. Either online structured questionnaires, shared via a Google Forms link on the class WhatsApp group, or paper-based versions, required approximately 20 minutes to complete.

### Data analysis

Data were analysed using IBM SPSS Statistics version 30.0 (IBM Corp., Armonk, New York [NY], United States [US]). Respondents were categorised as junior (1st year – 2nd year) or senior (3rd year – 4th year) nursing students. Comparisons were conducted between junior students from the two universities, as their data were collected at different phases of the COVID-19 pandemic. Senior students were not compared across universities because of the small sample size at University A.

Descriptive statistics (means, standard deviations and frequencies) were calculated for the total sample and subgroups. Group differences were examined using non-parametric tests: (1) the Mann-Whitney *U* test for continuous variables and (2) Pearson’s Chi-square test for categorical variables. Internal consistency of the scales was assessed using Cronbach’s alpha coefficients. All analyses followed the published scoring and interpretation guidelines for the respective instruments.

### Ethical considerations

To promote the well-being of all student participants, researchers informed students about the counselling and support services available at the universities during the study. This information was provided before participation, encouraging students to seek assistance for any issues related to psychological distress or anxiety about COVID-19, while ensuring their anonymity was safeguarded.

Ethics approval for the research was secured from the Research Ethics Committees of both universities involved (University of the Western Cape: HS20/10/17 and Cape Peninsula University of Technology: HWS-REC 2021/H21). Furthermore, permission to carry out the study was granted by the University registrars and the management of the nursing schools. All participants were given written information regarding the study and provided their informed consent prior to taking part. Participation was voluntary, allowing students the option to withdraw at any stage without facing any repercussions. The questionnaires were coded, anonymous, and securely kept ensuring confidentiality.

## Results

### Demographic profile and sample realisation

The final sample consisted of 589 respondents from University A (380, 64.5%) and University B (209, 35.5%). There were more juniors (443, 75.2%) than senior respondents (132, 22.4%) in the overall sample and the average age was 22.5 (± 4.6) years (*U* = 8.351, *p* < 0.001). There were more female (351, 79.2% and 96, 72.7%) than male respondents (86, 19.4% and 36, 27.3%) (*χ*^2^ = 3.47, *p* = 0.062) (Table 1).

**TABLE 1:** Demographic characteristics of the respondents (*N* = 589).

Characteristic	Junior (443, 75.2%)	Senior (132, 22.4%)	All	Test		*p*-value
				*U*	*χ* ^2^	
**Age**	-	-	-	8.351	-	< 0.001*
Mean	21.85	24.42	22.5	-	-	-
s.d.	± 4.05	± 5.20	± 4.6	-	-	-
**Gender**	-	-	-	-	3.47	0.062
Male:						
*n*	86	36	124	-	-	-
%	19.4	27.3	21.1	-	-	-
Female:						
*n*	351	96	456	-	-	-
%	79.2	72.7	77.4	-	-	-

Note: Values do not add up to 100%, it represents missing values.

*U*, Mann-Whitney *U* test; *χ*^2^, Pearson’s Chi-square test; s.d., standard deviation.

* , *p* < 0.05.

### Psychological distress

Overall, and across the groups, respondents reported mild levels of psychological distress, and respondents were most often classified as ‘likely to be well’ (285, 48.4%), followed by ‘mildly distressed’ (118, 20.0%). Senior respondents reported significantly higher levels of psychological distress than junior respondents (All: 20.89, ± 7.97; Junior: 19.69, ± 7.09; Senior: 24.83, ± 9.19) (*U* = 5.8, *p* < 0.001) (Table 2) and most often reported to be ‘likely to be well’ (40, 30.3%) or ‘severely distressed’ (36, 27.3%); as well as higher levels of ‘moderate’ (28, 21.2%) and ‘severe’ (36, 27.3%) distress compared to junior respondents (47, 10.6% each for ‘moderate’ and ‘severe distress’). Junior respondents were most often classified as ‘likely to be well’ (238, 53.7%), followed by ‘mildly distressed’ (94, 21.2%) (Table 2).

**TABLE 2:** Psychosocial distress, resilience and fear of coronavirus disease 2019 of junior respondents and senior respondents (*N* = 589).

Scale	Junior (443, 75.2%)	Senior (132, 22.4%)	All	Test (*U*)	*p*-value
**Psychological distress**	-	-	-	5.8	< 0.001*
Mean†	19.69	24.83	20.89	-	-
s.d.	± 7.09	± 9.19	± 7.97	-	-
Likely to be well:					
*n*	238	40	285	-	-
%	53.7	30.3	48.4	-	-
Mild distress:					
*n*	94	23	118	-	-
%	21.2	17.4	20.0	-	-
Moderate distress:					
*n*	47	28	76	-	-
%	10.6	21.2	12.9	-	-
Severe distress:					
*n*	47	36	86	-	-
%	10.6	27.3	14.6	-	-
**Resilience**	-	-	-	−1.6	0.106
Mean‡	71.43	69.32	71	-	-
s.d.	± 12.10	± 12.21	± 12.24	-	-
Low resilience:					
*n*	16	8	26	-	-
%	3.6	6.1	4.4	-	-
Moderate resilience:					
*n*	158	48	209	-	-
%	35.7	36.4	35.5	-	-
High resilience:					
*n*	214	62	285	-	-
%	48.3	47	48.4	-	-
**Fear of COVID-19**	-	-	-	−2.2	0.028*
Mean§	18.73	17.32	18.41	-	-
s.d.	± 6.86	± 7.14	± 6.94	-	-
No fear	0	0	0	-	-
Mild fear:					
*n*	241	85	333	-	-
%	54.4	64.4	56.5	-	-
Moderate fear:					
*n*	119	30	153	-	-
%	26.9	22.7	26	-	-
Severe fear:					
*n*	62	15	78	-	-
%	14	11.4	13.2	-	-

*U*, Mann-Whitney *U* test; COVID-19, coronavirus disease 2019; s.d., standard deviation.

* , *p* < 0.05.

† , maximum score: 50;

‡ , maximum score: 88;

§ , maximum score: 35.

### Resilience – Response to stress

Overall, the respondents reported high levels of resilience (71.0, ± 12.24, All: 285, 48.4%; Junior: 214, 48.3%; Senior: 62, 47%) with only a small percentage reporting low levels (All: 26, 4.4%; Junior: 16, 3.6%; Senior: 8, 6.1%) (Table 2). Junior respondents reported high (71.43, ± 12.10) and senior respondents moderate (69.32, ± 12.21) levels of resilience (*U* = –1.618, *p* = 0.106) (Table 2).

### Fear of COVID-19

Overall and across the groups, respondents reported mild levels of fear of COVID-19 (All: 18.41, ± 6.9; Junior: 18.73, ± 6.86; Senior: 17.32, ± 7.14) (*U* = –2.195, *p* = 0.028) (Table 2). In addition, junior respondents from University A (19.20, ± 6.77) reported higher levels of fear of COVID-19 than junior respondents from University B (16.65, ± 6.90) (*U* = –2.894, *p* = 0.004).

## Discussion

The aim of this study was to investigate psychological distress and resilience among junior and senior undergraduate nursing students from two South African universities. The age difference between junior groups and senior groups was statistically significant, which partly reflects differences in the proportion of senior students enrolled across the two universities.

Overall, students reported only mild psychological distress, although senior students experienced substantially higher levels than junior students. This pattern aligns with previous research showing that senior students face greater academic and clinical demands, including responsibility for more complex patient care and preparation for professional practice (Dias et al. 2024; Mohamed et al. 2024). Senior students’ exposure to higher clinical workloads, combined with the ongoing challenges of the COVID-19 pandemic, likely compounded stress. Comparable findings have been reported internationally, where senior students experience heightened anxiety during clinical placements and transitions to practice (Lavoie-Tremblay et al. 2022).

Interestingly, despite experiencing greater psychological distress, senior students reported lower levels of fear of COVID-19 compared to juniors. This may be explained by greater clinical exposure and competency in infection prevention, which enhanced confidence and reduced fear. Prior studies confirm that clinical experience, organisational support and COVID-19–specific training mitigated anxiety among nurses and students (Labrague & De los Santos 2020; Labrague & De los Santos 2021; Rahman 2022). At both universities, the presence of clinical mentors and opportunities to participate in vaccination programmes may also have served as protective factors. This highlights the value of structured mentorship and experiential learning in reducing fear and building coping capacity.

The difference in fear of COVID-19 between universities appears related to the timing of data collection. Students assessed earlier in the pandemic reported higher fear, consistent with studies showing that uncertainty, misinformation and limited vaccine access amplified anxiety during the initial phases (Awijen, Ben Zaied & Nguyen 2022; Zhao et al. 2023). By contrast, those assessed later, when vaccines were available and restrictions eased, reported less fear, in line with evidence linking knowledge, perceived risk, and public health messaging to student anxiety (Alsolais et al. 2021; Jarvis et al. 2021). This underscores that student mental health research should account for broader contextual factors such as timing, public perceptions and evolving pandemic conditions.

Resilience emerged as a consistent protective factor across both groups, with most students demonstrating high levels. This finding mirrors research indicating that, despite pandemic-related stress, nursing students often maintain moderate to high resilience (Labrague 2021; Savitsky et al. 2020). The availability of institutional support such as counselling services may have contributed to these results. Importantly, resilience is not merely a personal trait but can be cultivated through education and supportive environments (Lopez et al. 2018). For nursing education, this underscores the need to embed resilience training into curricula, through strategies such as reflective practice, stress management skills and peer support programmes (Sherwood 2024).

At the same time, nursing remains a demanding profession characterised by chronic stress and risk of burnout (Goudarzian et al. 2024). While resilience enabled students to cope with acute challenges during the pandemic, ongoing exposure to poorly managed stressors may compromise well-being and professional sustainability (Mbunge 2020). Long-term strategies are therefore needed to support nurses across the career trajectory, from student training to professional practice.

### Recommendations

Regarding nursing education and policy, research shows that nursing programmes should include ways to build resilience. This can involve structured peer support, stress management workshops, and peer to peer mentorship programmes to help students deal with clinical and academic challenges. At the policy level, it is essential to include resilience support in national nursing education plans in South Africa. This will help ensure that nursing students are ready to face the pressures of their profession and public health crises.

### Limitations

The timing of data collection across different universities, which happened during various phases of the pandemic. Changes in perception, vaccine availability and infection rates may have affected how fearful nursing students were of COVID-19 and their levels of distress, making it hard to compare the groups. We also acknowledge that the two groups differ in size and that the findings should therefore be interpreted with caution.

## Conclusion

This study shows that nursing students were able to demonstrate resilience in the face of an unusual stressor, such as the COVID-19 pandemic, with juniors and seniors displaying different patterns of distress and fear. While resilience was generally high, the elevated distress among seniors underscores the ongoing need for targeted psychological support. Building resilience into nursing education is essential for preparing students to manage future crises and for sustaining a healthy, adaptable nursing workforce.
