# Corrigendum: Responding to extreme stress: Resilience and psychological distress in undergraduate nursing students in the Western Cape, South Africa

**DOI:** 10.4102/curationis.v49i1.2950

**Published:** 2026-07-14

**Authors:** Jennifer Chipps, Regis R. Marie Modeste, Amanda Cromhout, Ilze Steenkamp

**Affiliations:** 1 School of Nursing, Faculty of Community and Health Sciences, University of the Western Cape, Cape Town, South Africa; 2 Department of Nursing and Midwifery, Faculty of Medicine and Health Sciences, Stellenbosch University, Cape Town, South Africa

In the published article, Chipps, J., Marie Modeste, R.R., Cromhout, A. & Steenkamp, I., 2026, ‘Responding to extreme stress: Resilience and psychological distress in undergraduate nursing students in the Western Cape, South Africa’, *Curationis* 49(1), a2845, https://doi.org/10.4102/curationis.v49i1.2845, there was an error in affiliation 2.

Instead of:

Department of Nursing and Midwifery, Faculty of Health and Wellness Sciences, Stellenbosch University, Cape Town, South Africa

It should be:

Department of Nursing and Midwifery, Faculty of Medicine and Health Sciences, Stellenbosch University, Cape Town, South Africa

The authors apologise for this error. The correction does not change the study’s findings of significance or overall interpretation of the study’s results or the scientific conclusions of the article in any way.

